# First Report of *Ganoderma ryvardenii* causing Basal Stem Rot (BSR) disease on oil palm ( *Elaeis guineensis* Jacq.) in Ghana

**DOI:** 10.12688/f1000research.161972.2

**Published:** 2025-07-07

**Authors:** Emmanuellah Lekete-Lawson, Grace C. van der Puije, Enoch A. Osekre, Frank K. Ackah

**Affiliations:** 1 Department of Crop Science, University of Cape Coast College of Agriculture and Natural Sciences, Cape Coast, Ghana; 2 CSIR-Oil Palm Research Institute, Kade, Eastern Region, Ghana; 3 Department of Crop and Soil Sciences, Kwame Nkrumah University of Science and Technology College of Agriculture and Natural Resources, Kumasi, Ghana

**Keywords:** Ganoderma disease, Basal Stem Rot, oil palm, Ghana, phylogenetic

## Abstract

**Backgrounds:**

Oil palm ( *Elaeis guineensis* Jacq.), is the most significant and highest-yielding crop among oil-producing crops worldwide. In 2020/2022, Basal stem rot (BSR) disease was observed in six oil palm growing Districts in Ghana.

**Methods:**

Field study and laboratory analysis were conducted. A random sampling technique was used to select five plantation blocks from each District. Single-point disease assessments were done using Standard Operating Procedure (SOP) with a severity scale of 0-4. Molecular assays were performed on each sample using nucleic acid as a template. ITS and GanET sequence analysis were performed along with the formation of a phylogenetic tree using the FASTA algorithm with the Fungus database from EBI and NCBI GenBank. Koch’s postulate was followed to confirm the disease.

**Results:**

The disease incidence was 11.3 % with the highest severity score of 4. Basal Stem Rot (BSR) is characterised by progressive stem decay coupled with the formation of large, perennial, woody basidiocarps. The average measurement of 2-65 cm in diameter on infected palms. Culture colonies were white, striated, undulating, woolly-cottony, and creamish pigment on the reverse, depicting attributes of *Ganoderma* fungus. Molecular confirmation was done by combining the ITS sequence of top matches of >97% to members of the genus *Ganoderma*, >98% and 99.3% identity to three sequences of *Ganoderma* sp. (HM138671; HM138670 and HM138672) generated from strains assigned to *Ganoderma ryvardenii* and compared with 132 published sequences of *Ganoderma* isolates.

**Conclusion:**

This study presents the first report of *Ganoderma ryvardenii* causing BSR disease on oil palm in Ghana, potentially the first in West Africa, and second in Africa. Notably, the pathogen was previously first reported to cause similar disease on oil palm in Cameroon, highlighting its emerging threat to oil palm production in the Sub-Saharan African region.

## 1. Introduction

Oil palm ( *Elaeis guineensis* Jacq), is considered a worldwide pre-eminent and highest-yielding edible oil crop among other oil-producing crops and has since inspired many economically emergent nations including Malaysia, Indonesia, India, who engaged in oil palm production ( Ghulam, 2022). Although oil palm is known to have originated from Africa, only 73.8 million metric tons, representing 3%, was produced by the continent in 2021/22 ( Biney et al., 2024), with West Africa making 4% ( FAOSTAT, 2022). Ghana’s oil palm production is about 0.5 million metric tons annually ( Biney et al., 2024; Sasu, 2023), representing a tiny portion of the global total despite being endowed with high-yielding varieties. Although Ghana can produce more oil palm, challenges including pests and diseases as well as climate variability prevent it from expanding ( Zutah et al., 2024). Nonetheless, by addressing these challenges, Ghana will improve its oil palm production and ensure less dependency on food imports.

Among the diseases reported on oil palms in Ghana is Basal Stem Rot (BSR), which is suspected to be caused by the white rot fungus called *Ganoderma* *. Ganoderma* sp. is a basidiomycetes fungus and has been reported to be the primary cause of BSR disease. It is the most devastating and destructive disease of oil palm in tropical regions and causes substantial economic losses up to 43% biannually to oil palm producers ( Khoo & Chong, 2023). According to Karunarathna et al. (2024), BSR disease can cause yield reductions of up to 80% in severely affected plantations, with an estimated annual loss exceeding 50 million metric tons. It is, therefore, projected that if the necessary actions are not taken, BSR disease can wipe out close to 860,610 ha of mature oil palm plantations in Malaysia by 2030 ( Olaniyi & Szulczyk, 2020).

Several *Ganoderma* spp. viz. *G. applanatum,* *G. boninense*, *G. zonatum*, *G. chalceum*, *G. lucidum*, *G. miniatocinctum,* *G. ryvardenii*, *G. pseudoferreum* and *G. tornatum* have been linked to BSR disease on oil palm by early researchers ( Midot et al., 2019; Kumar et al., 2022; Kinge & Mih, 2011; Turner, 1981). Studies conducted by Chakrabarti et al. (2023) and Mohd Rakib et al. (2014) confirmed *G. zonatum* as the most prevalent fungal pathogen responsible for BSR and upper stem rot (USR) disease in oil palms, accounting for 71.7% of the total infected samples collected. Moncalvo (2000) also reported that *G. boninense* is the most virulent species, causing a high incidence of BSR disease in oil palm. Other researchers also confirmed these observations ( Bharudin et al., 2022; Khoo & Chong, 2023; and Susanto et al., 2005).

Nonetheless, a comprehensive study conducted in the littoral and southwestern regions of Cameroon, by Kinge and Mih in 2011, to identify *Ganoderma* species associated with basal stem rot (BSR) disease of oil palm. During the survey, these researchers encountered a previously unclassified Ganoderma species which morphological and molecular analyses revealed to be distinct from all the known species ever reported To determine its identity, a Morphological and molecular characterisation of the ITS rDNA locus was done which revealed variation of ellipsoidal basidiospores with narrowly truncate apices to differentiate it from its close relatives like G. *steyaertanum* and G. *boninense* clade but as a standalone strongly supported lineage. . Expressing their admiration for its distinctiveness, researchers named it *Ganoderma ryvardense*, in appreciation of the extraordinary mycologist Leif Ryvarden, whose outstanding work in African fungal taxonomy cannot be ignored.

All these reports indicated that BSR disease in oil palm is associated with varied pathogenic species of *Ganoderma* fungi. To address the issue concerning species variability, and accurately identify and confirm the actual Ganoderma species causing BSR disease at a particular location, many researchers resorted to more accurate, faster and more sensitive molecular diagnostic approaches instead of the old traditional culture-based assay, which is more labour-intensive and time-consuming ( Seemanti et al., 2022). Therefore, for effective management strategies, this study adopted these molecular diagnostic techniques to effectively and accurately identify and confirm the actual Ganoderma species involved in causing BSR disease on oil palm in Ghana.

## 2. Materials and methods

### 2.1 Study area

Disease assessment was conducted in 30 oil palm growing communities in six districts in Central, Eastern and Western Regions of Ghana, two districts per region and five farming communities per district. For the purpose of this study, the selected districts surveyed were renamed as K29B, K37-1, K31-1, K37-2, K30, and K4. The K29B and K37-1 represent Denkyembour and Fateakwa districts from the Eastern region, K31-1 and K37-2 represent Nzema West and Mpohor districts from the Western region, while K30 and K4 represent Twifo-Atti Morkwa and Gomoa East districts from Central Region.

### 2.2 Climate of the study area

The districts visited in Eastern region, lie within latitudes 6°.10871′ N to 6°.38431′ N and longitudes 0°.35047′ W - 0°.30′ W and 0°10′E to 0°30 E. They cover land areas ranging from 1500 km ^2^ to 2050 km ^2^ of the region. In the Western region, the two districts lie within latitudes 4.8520°N to 5.2 609°N and -2.2377° W to 1.5021°W and covered land areas ranging between 1,200 km ^2^ to 6,231 km ^2^. Twifo-Atti Morkwa (K30) and Gomoa East (K4) districts from the Central region lie within latitudes 5°3.209′ N to 5°7.891′ N and longitudes 1°23.200′ W - 1° 54.360′ W. They cover land areas ranging from 1,160 km ^2^ and 5,231 km ^2^ of the region. All these regions are located within the semi-deciduous forest zone in Ghana. The areas are characterized by a double maxima rainfall pattern followed by a prolonged dry season. The minimum temperature during the study period (October 2022 to February 2024) ranged between 22.6°C and 25.2°C and the maximum varied between 31.4°C and 36.0°C. The relative humidity varied from 45% to 75%.

### 2.3 Field observation and assessment

Intensive BSR disease scouting was carried out based on the single-point disease assessment using the standard operating procedure (SOP) of the *Ganoderma* census published by the Malaysian Palm Oil Board (MPOB, 2014). The process was done by counting the number of infected trees using a block of a hundred trees along a diagonal on each plantation for proper disease representation. A total of 3000 palms were assessed, 100 palms per plantation.

### 2.4 Percentage disease intensity

Disease incidence was computed using modified version of Vicent’s formula ( Lekete-Lawson *et al*., 2024).

$$\%\mathrm{BSR}\;\text{Disease Incidence}\;\left(\mathrm{DI}\right)\mathrm{on}\;\text{the field}=\frac{\text{Number of}\;\mathrm{BSR}\;\text{infected oil palm}\;\mathrm{per}\;\text{Plantation}}{\text{Total number of selected oil palm}\;\mathrm{per}\;\text{Plantation}}\times 100$$

Disease severity was scored on a scale of 0-4 ( Lekete-Lawson *et al*., 2024)

where, 0 = no symptom, and 4 ≥ 9 Maximum number of fruiting bodies per palm or foliar symptoms > 4 fronds/palm. This is demonstrated in the table below.

**Guide**

**Table:** Disease severity index for Ganoderma infection (BSR) of oil palm.

Severity score	No. of BSR fruiting bodies/foliar symptoms/plant	Percent diseased palm
0	No BSR fruiting body/no foliar symptoms	Healthy plant or no disease
1	1–3 BSR fruiting body/foliar symptoms on two fronds	1–25%
2	4–6 (BSR) fruiting body/foliar symptoms on three fronds	26–50%
3	7–9 (BSR) fruiting body/foliar symptoms on four fronds	51–75%
4	>9 (BSR) fruiting body/foliar symptoms on > four fronds	76–100%

### 2.5 Sampling and isolation

**Figure 1.**
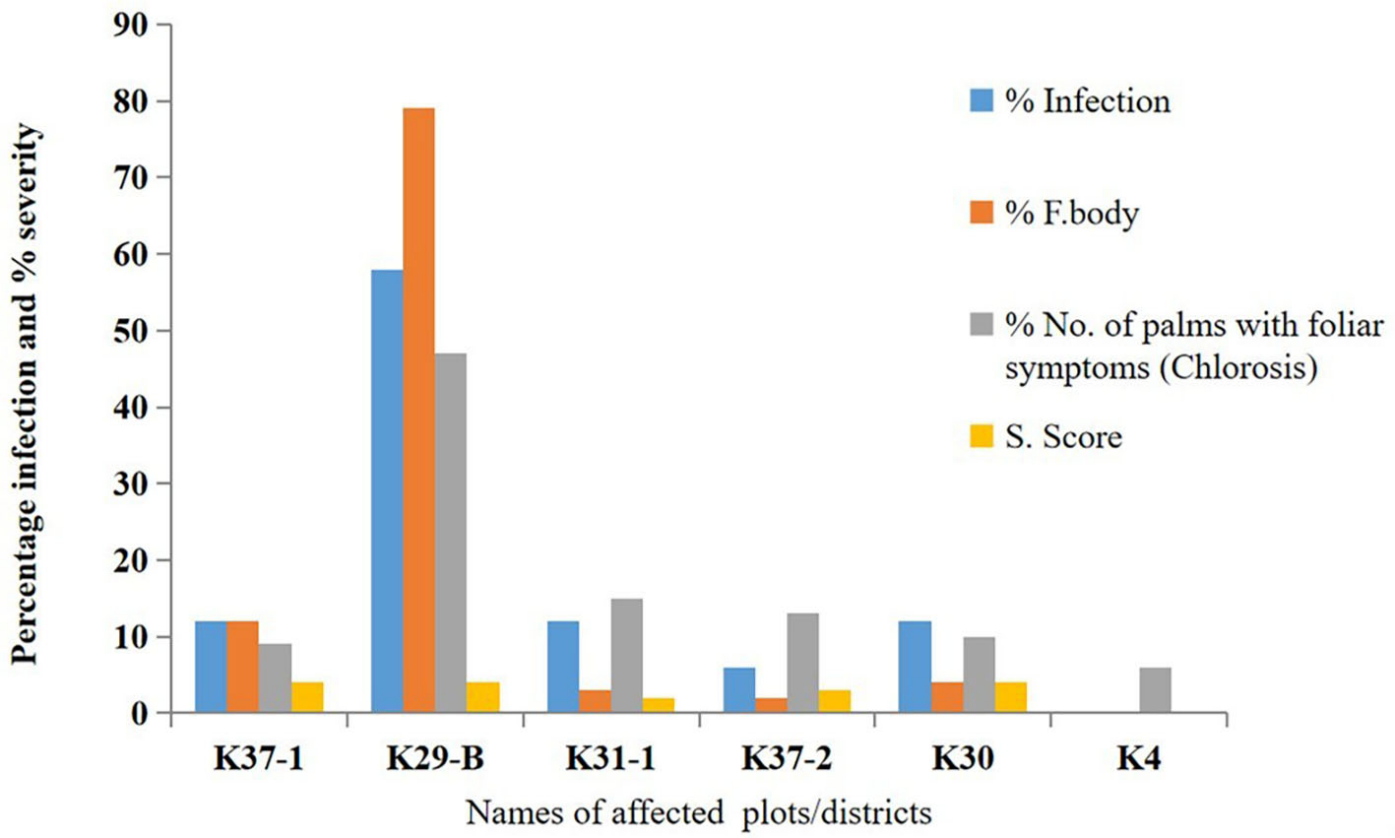
Prevalence of BSR disease in selected Districts in Ghana.

A total of ninety (90) symptomatic samples, comprising of basidiocarps (fruiting bodies) and infected rotten plant tissues were collected from infected oil palms across all the three agro-ecological regions under investigation ( Figure 1).

### 2.6 Fungal culturing and isolation

Media compositions were as follows: PDA for one litre of distilled water included: 39 g of Pre-mixed, Dehydrated PDA powder (Supplier: Thermo Fisher Scientific, Catalog code: CM0139B amended with 3 g of peptone (supplier: Clinichem Ltd, catalogue code: 70161), pH: 5. Malt extract agar (MEA: supplier: Thermo Fisher Scientific, Catalogue code: CM0059B); composition per liter of distilled water: 30 g of malt extract, 3 g of peptone, and 15 g of agar (Supplier: Millipore Sigma, Catalogue number: 05040); pH: 5.6. The diseased samples collected from both the infected oil palm trunk and the Basidiocarps were cut into approximately 1 cm pieces with a scalpel blade and rinsed three times in sterilized distilled water, surface sterilized in a 10% sodium hypochlorite (solution, and blotting on tissue paper) ( Goh et al., 2014). The samples that were sterilized were aseptically inoculated onto PDA powder (Supplier: Thermo Fisher Scientific, Catalog code: CM0139B) and incubated at 28±1 °C for four days and later sub-cultured on Malt Malt extract agar (MEA: supplier: Thermo Fisher Scientific, Catalogue code: CM0059B) till pure cultures of isolated fungi were obtained. The pure fungal cultures were maintained on Malt Extract agar (MEA) Malt extract agar (MEA: supplier: Thermo Fisher Scientific, Catalogue code: CM0059B) as stock cultures in Petri dishes in dark conditions at room temperature (28 ± 1°C) for further analysis.

### 2.7 Morphological identification

Macro-morphological identification and confirmation were done using colony characteristics (shape, colour and texture of the mycelia) and micrograph description. Cultures from various plantations were grouped based on their morphological resemblances. There were three replications per isolate. Sporulation was assessed on glass slides by mounting a small portion of mycelia in sterilized distilled water with a blue stain and observed under a Leizer microscope of lens magnification of 40X. Mycelia, obtained from the various samples, were preserved in the biological incubation chamber (Thermostatic Cabinet ST 1 POL-EKO2; ST 1/1/1, Manufacturer: **POL-EKO **. Registered trademark ( ^®^^,^™): POL-EKO ^®^ sp.k of POL-EKO-APARATURA sp.j.) at 20 °C for 12 days, for pathogenicity tests and other clinical analyses.

### 2.8 Molecular identification and confirmation

Infected BSR samples, fruiting bodies and isolated fungi were taken to the CABI Microbial Identification Service, Bakeham Lane, UK for Molecular identification and confirmation.

**2.8.1 Procedure as follows**

All original samples ( *Ganoderma* fruiting bodies and isolated fungi) were tested for purity. Molecular assays were conducted on all samples with nucleic acid as the template. A total of 29 basidiocarps and 24 Ganoderma pure cultures were investigated. For DNA extraction, a proprietary formulation, microLYSIS ^®^-PLUS (MLP) from Microzone, UK, was used, for rapid thermal cycling to ensure efficient cell lysis and DNA release. Polymerase Chain Reaction (PCR) was used to amplify the rDNA in vitro after DNA extraction. The DNA quality of the PCR products was then evaluated. The samples were electrophoresis using a 2% agarose gel (Supplier of agarose gel: Thermo Fisher Scientific, Catalogue Number: UltraPure™ Agarose, 500 g – 165005001) and GelRed-stained (supplier: Biotium, Inc., Catalogue Number: GelRed ^®^ Nucleic Acid Stain, 10,000X in water - 41003/41003-1). An additional PCR purification step was performed to eliminate excess dNTPs, primers, polymerase, and other constituents of the PCR mix, in order to obtain an extremely pure DNA template for sequencing. PCR amplicons were later characterised by sequencing with the BigDye ^®^ Terminator v3.1 kit from Applied Biosystems (Life Technologies, UK,) and a pair of primers: ITS1 (TCC GTA GGT GAA CCT GCG G) and ITS4 (TCC GCT TAT TGA TAT GC GTAC) ( Gašparcová et al., 2017). The basidiomycete-selective primer GanET (GCGTTACAT GAG CGCAATACAA) was also used to prevent the potential co-amplification of contaminant non-basidiomycetous fungi.

Sequencing reactions were carried out with the use of fluorescently tagged chain terminator dNTPs. Sample processing was executed with the AB 3130 Genetic Analyzer for the examination of the sequence of nucleotide bases (adenine, guanine, cytosine, and thymine) in the DNA oligonucleotide. Preliminary identification was made by matching the sequenced data with the sequences in the European Molecular Biology Laboratory (EMBL) database via the European Bioinformatics Institute (EBI). For verification, the samples were re-identified by ITS sequence analysis using the FASTA algorithm and the Fungus database from EBI and the BLAST algorithm with the NCBI standard database (Report on molecular work: https://figshare.com/s/86864d9d08393658fb20) (Accessed: 07.02.2025).

**2.8.2.0 Phylogenetic analysis**

**2.8.2.1 Sequence alignment**

Sequences of newly identified species were analysed using standard BLAST searches in GenBank: [ Lekete-Lawson, (2025a), ( https://doi.org/10.6084/m9.figshare.28389083)] to identify more similar sequences. In addition to sequences obtained in this study, all sequences used for phylogenetic analysis were retrieved from GenBank: ( ftp://ftp.ncbi.nlm.nih.gov/pub/agarwala/indexed_megablast). The sequence multiple alignments were done using the online version of MAFFT (v7.511) https://mafft.cbrc.jp/alignment/software/ and open access online version of Geneious Bioinformatics Software for sequence Data analysis R9 v. 2019.2.3 ( https://www.geneious.com/): Rozewicki et al. (2019) manually formatted with Clustal-Omega 1.2.4 (open access available: http://www.clustal.org/omega/) pairwise sequence alignment tools to minimise gaps and ensure proper alignment.

### 2.9 Pathogenicity test (Koch’s postulate)

For the purpose of this study, three-weeks-old healthy germinated palm seeds of commercial standard crosses of (Dura × Pisifera, (D × P)) obtained from Ghana Sumatra Ltd were used for the pathogenicity test. A volume of 250 ml Beatson glass jars (R3/83 mm 4 Doz) containing a mixture of planting media (black soil, bio-cha, and treated plant biostimulant) at 2:2:1 100 g per glass was used. The jars were autoclaved for 45 minutes at 121°C. The mixtures were left to cool for three days. After three days, healthy germinated seeds with uniform growth were sown into the glass jar containing the sterilized growth medium for artificial inoculation ( Goh et al., 2014). A total of Hundred and forty-four (144) germinated palm seeds germinated palm seeds (three treatment and control, 6 seeds per replicate) were arranged in a randomised complete block design (RCBD) in the growth chamber and maintained for one week under laboratory conditions at CSIR-OPRI Plant Pathology laboratory, (6°.10871′N, 0°.35047′W and 0°10′E) with relative humidity and temperature regulated at 70 % and 28±1°C, respectively.

**2.9.1 Inoculum preparation**

Research has shown that *Ganoderma* species are wood-decomposing fungi that thrive on decaying wood, which provides essential nutrients and moisture ( Kumar et al., 2022; Mangaiha et al., 2019; Chang et al., 2002). Thus, techniques by Angel et al. (2021) and Bivi et al. (2016), with slight modifications, were used in *Ganoderma* inoculum preparation using Woodblocks made from *Triplochitan scleroxylon* (Wawa), which was previously tested in preliminary studies (Anonymous, 2024-unpublished) to be an effective growth medium for *Ganoderma* fungi. The Wawa Woodblock (WWBs) incubation time was 15 days ( Breton et al., 2005, 2006).

**2.9.2 Artificial inoculation of germinated oil palm seeds**

Inoculation of germinated seeds was performed in 250 ml Beatson glass jars (R3/83 mm 4 Doz) with WWBs previously colonized by *Ganoderma* isolates. Glasses (components) were incubated according to the method described by Lo et al. (2023) with slight modification. The set-up was maintained under lab conditions with relative humidity and temperature regulated at 70% and 28±1°C, respectively. The infection process was monitored and recorded daily. Germinated seed nuts with WWBs without inoculum served as a negative control.

Re-isolation of inoculated fungi from the destructive samples was performed following the techniques by Agrios (2005) and Idris et al. (2006). The roots were treated and plated on Malt extract agar and subcultured until a pure culture was obtained and identified by Lo et al. (2023).

**2.9.3 Symptoms recording**

Disease development based on external and internal symptoms was recorded every three days. External symptoms were based on visual estimation of the proportion of tissues damaged by inoculated fungi, using the scale established by Breton et al. (2006). Internal symptoms were recorded by splitting the inoculated seed nuts in two cross-sections and through the root. Other symptoms which were not visible were confirmed using molecular tools. All the signs and symptoms recorded were used for disease rating.

**2.9.4 Disease rating**

Disease rating *in vitro* was computed as:

$$\%\mathrm{BSR}\;\text{Disease Incidence}\;\left(\mathrm{DI}\right)\;\mathrm{per}\;\text{germinated seed}\;\mathrm{nut}=\frac{\text{Number of infected oil palm seed nuts}}{\text{Total number of inoculated seed nuts}}\times 100$$

### 2.10 Data analysis

The results for interpreting pathogenicity tests were based on the percentage of infection on test seed nuts. These were subjected to an ANOVA, and treatment means were separated with the least significant difference (LSD) at 5% after rating (R-Software data analysis: Lekete-Lawson, E. (2025a) https://figshare.com/authors/Emmanuellah_Lekete-Lawson/20618918). The data on different parameters were also compiled and analysed using the R-Stat version 2021 statistical package ( R Core team (2021) https://www.R-project.org, accessed 10/8/2024). The LSD at 1% was also used to separate the means of treatment fungi and the control.

## 3. Results

### 3.1 Field observation and percentage infection

Out of 3000 palm trees assessed, 341 palms were identified as *Ganoderma-*infected palms representing 11.3% of disease incidence. However, the level of infection differs. The number of fruiting bodies (basidiocarps) counted on each infected palm varied between 2 and 274, and severity scores were between 2 and 4, with 4 being the highest recorded on the 9-year-old palm. District K29-B in the Eastern region showed the highest percentage of infection, with 161 infected palms and 274 basidiocarps. District K4 has the least, with 2 infected palms and 4 basidiocarps representing a severity score of one.

This study revealed a higher infection rate in the Eastern region compared to the other two regions surveyed. Figure 1 shows the total number of palms affected and the severity score range of BSR disease in the selected oil palm growing Districts in Ghana.

### 3.2 Morphological characteristics of Ganoderma symptoms

**Figure 2.**
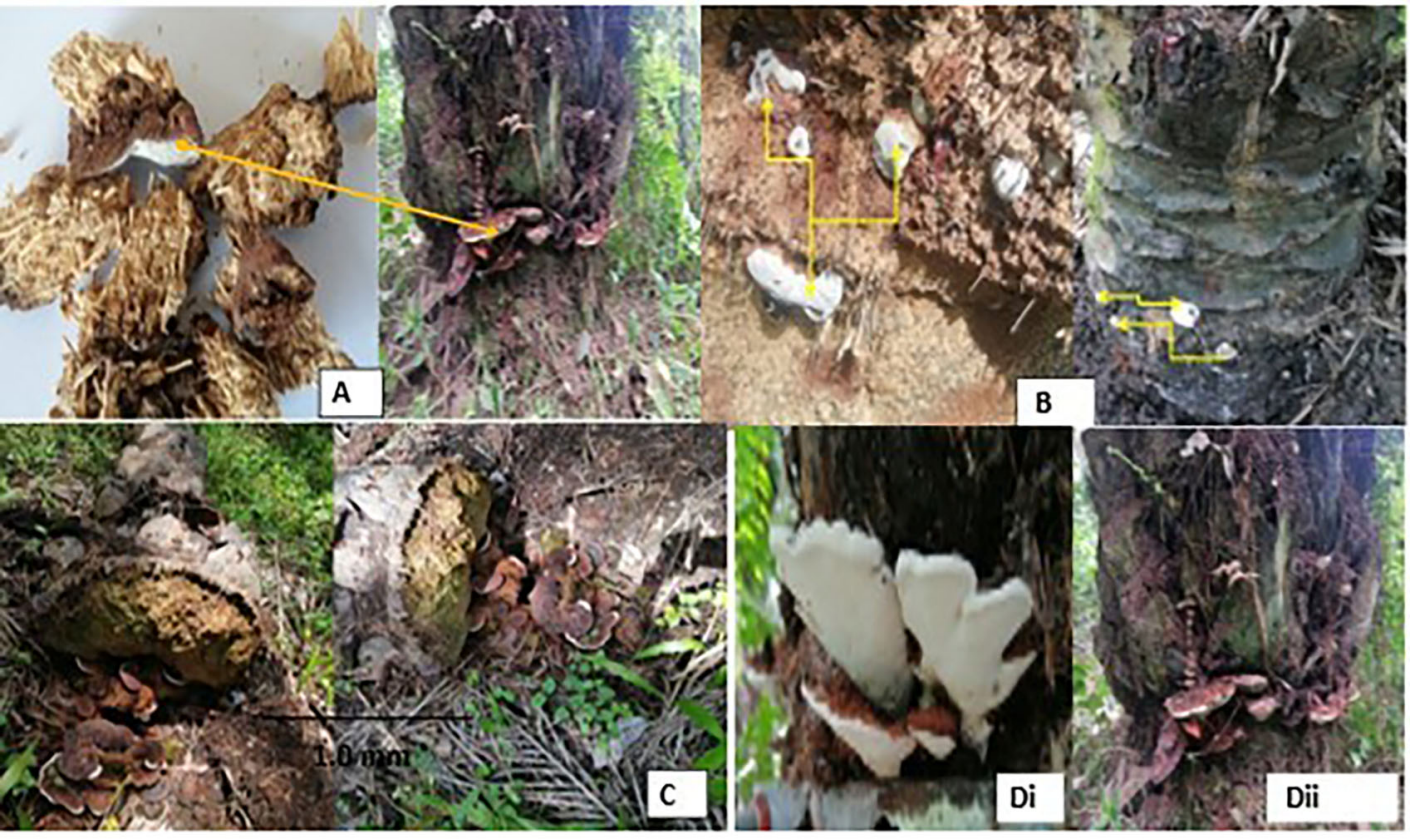
Ganoderma-infected palms: (A) symptomatic samples for isolation, (B) Initial stage of infection and pinhead of the Ganoderma mushroom, (C) Fractures and collapsed Ganoderma infected palm, (Di) Upper Stem Rot infection (Dii) Basal Stem Rot infection.

Basal stem rot disease observed on the fields depicts the basidiocarps which first appeared as small white buttons ( Figure 2B) of tissue on the stem and later developed into a bracket shape mainly on the base ( Figure 2Dii) or at the upper side ( Figure 2Di) causing Basal Stem Rot (BSR) or Upper Stem Rot (USR) disease. These basidiocarps are woody, perennial and dimidiate; their cap (pileus) is concave, and some are circular with rough and irregular margins/blades (lamellae/gills). Pileus measured from 4-17 cm in diameter ( Figure 2Di); and 7 cm to 19 cm in length. Surface glabrous; crisped; shiny; dry and smooth. Their upper surfaces also took on a variety of white to yellowish-brown colours and was with concentric zonations ( Figure 2Dii). Also, the underside/gills are white ( Figure 2), gills adnexed, crowded (8-14 attached lamellae) with 8 series of lamellae, narrow and crisped. Most of the BSR basidiocarps lacked a stipe. However, in some upper-stem rot mushrooms, a short, wide, and noticeable stipe was present ( Figure 2Di). This stipe was lateral, compressed, and uniform in width, with a bulbous base and distinct longitudinal striations. The hymenophore is woody and dark-brown with no volva. No basidiospores, basidia, and basidioles were observed. The substrates were observed in live mature oil palms ( Figure 2).

Many of the heavily infected palms were seen full of basidiocarps growing from the base ( Figure 2 A, B, C & Dii); some deteriorated and others fractured and collapsed ( Figure 2C).

### 3.3 Macromorphological Characteristics of culture of *Ganoderma* fungi

**3.3.1 Isolation and identification of causal agent(s) of the BSR disease on oil palm**

**Figure 3.**
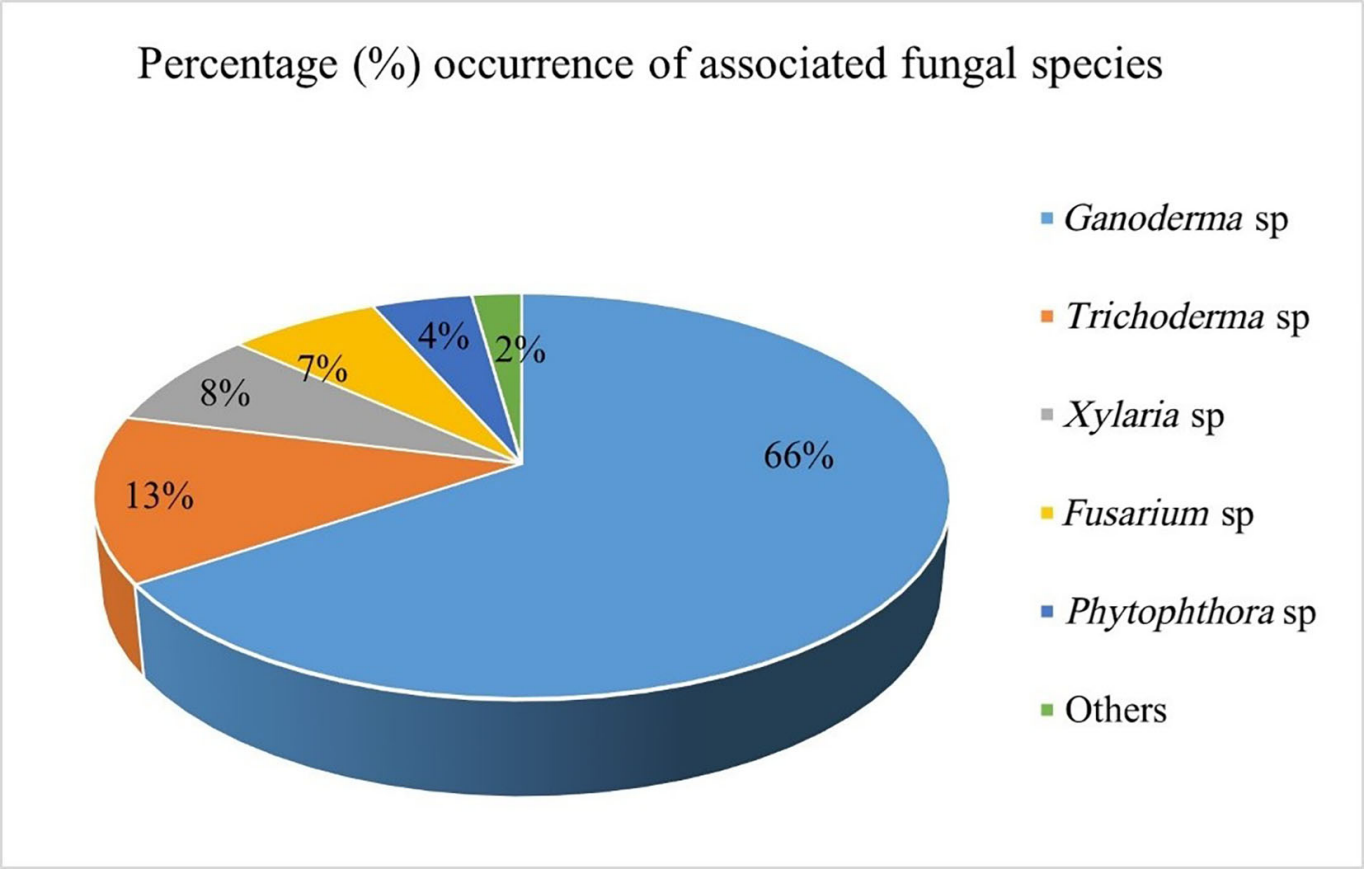
Common fungal species associated with Ganoderma-infected palms.

**Figure 4.**
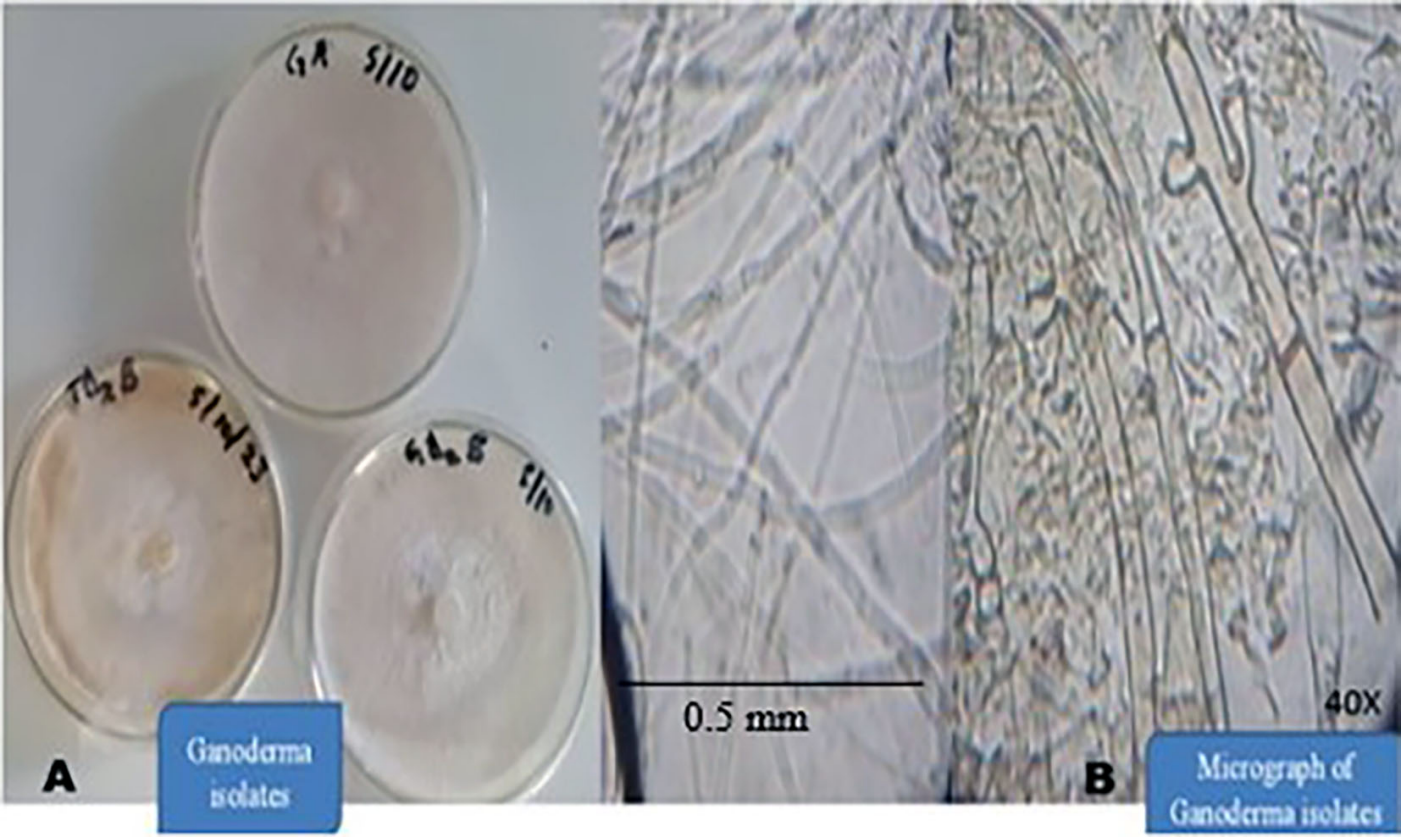
A) Pure culture of suspected *Ganoderma* sp. B) Micrograph of the isolates (image magnified 40X).

Out of 90 symptomatic samples collected from the field, 60 of them were screened for fungal isolation. 29 categories of fungal cultures were obtained and identified, 24 of them were identified as suspected *Ganoderma* fungi, and grouped into three (GA, GD2B and TD2B) ( Figure 4). Other fungi and oomycetes isolated and identified include *Trichoderma, Xylaria, Fusarium*, *Phytophthora*, other minor ones also include (moulds; *Rhizopus*, *Aspergillus* spp.). These fungal isolates were grouped based on their macromorphological characteristics and micrograph features. They were further ranked based on their taxonomy and percentage of occurrences ( Figure 3).

Overall, suspected *Ganoderma* fungi were identified 24 times, representing 66% of the total isolates. This was followed by *Trichoderma* sp. (13%) with saprophytic fungi (others) recording the least (2%). These suspected *Ganoderma* fungi were also categorised into three based on colony similarities in culture media, growth rate and their place of origin. Isolates GA from the Eastern, GD _2_B from the Central and TD _2_B from the Western regions ( Figure 4).

All cultures in Figure 4A were produced simultaneously. GA had the fastest growth rate, followed by TD _2_B and GD _2_B. These three groups of the Ganoderma isolates produced white, velvety, fluffy and cottony colonies on Malt extract agar medium ( Figure 4A). GA colonies appeared white, stranded, cottony and fluffy ( Table 1). GD _2_B produced white and stranded mycelium, velvety with concentric cottony and hyphal-knot structure ( Table 1). TD _2_B appeared white, fluffy, cottony and velvety but turned yellowish-brown as it progressed ( Figure 4). Their mycelium micrograph also shows hollow, septate and clamp connections ( Figure 4B) when viewed under a compound microscope with a magnification of 40X. No spore was seen on the artificial medium. Table 1 shows macromorphological features of the three groups of *Ganoderma* fungi isolated.

**Table 1.** Macromorphological features of pure cultures of suspected *Ganoderma* isolates.

Isolate group	Classification	Colony characteristics
GA	*Ganoderma* sp.	The colonies are whitish and radial, mycelium covers the entire Petri dish, creating dense and fluffy colonies in the centre. Colonies are continuous, radial, and have a cotton-like texture with snow-white (niveus) and later whitish (albidus) colours. They grow radially and form thinner and denser zones, with the centre becoming yellowish.
GD2B	*Ganoderma* sp.	The colonies are whitish, have a cotton-like texture and a strong leather-like appearance, hard to cut with a scalpel. They have slightly fuzzy margins and a snow-white (niveus) colour similar to GA isolate. After 12 days of inoculation, the surface becomes floccose with flaky mycelial formations, turning snow powder-like snow-white (niveus) and whitish (albidus) colours. stranded mycelium, velvety with concentric cottony and hyphal-knot structure.
TD2B	*Ganoderma* sp.	Colonies are whitish, dense, and thick after 8 days of inoculation. They take a yellowish shade, form radial depressions, and become dense and fluffy. Colony turns yellowish/brownish at the margin after covering the entire Petri dish.

*Legend: Abbreviations: sp. – species; *GA = Ganoderma fungi from Eastern region; *GD2B = Ganoderma fungi from Central region; *TD2B = Ganoderma fungi from western region.

### 3.4 Confirmation of pathogenicity test of isolated fungi

**3.4.1 Source of inoculum**

The study showed that the three isolated *Ganoderma* fungi (GA, TD _2_B and GD _2_B) were able to colonise the inoculated WWBs. The first sign of growth was observed after seven days of inoculation with GA isolate, TD _2_B isolates after 12 days and GD _2_B appeared after 16 days of inoculation. The total incubation time of the WWB inoculum was 17 days. The growth chamber was maintained at a temperature of 28±1°C and relative humidity of 60-75% suitable for the fungal growth.

**3.4.2 *In vitro* artificial inoculation**

**Figure 5.**
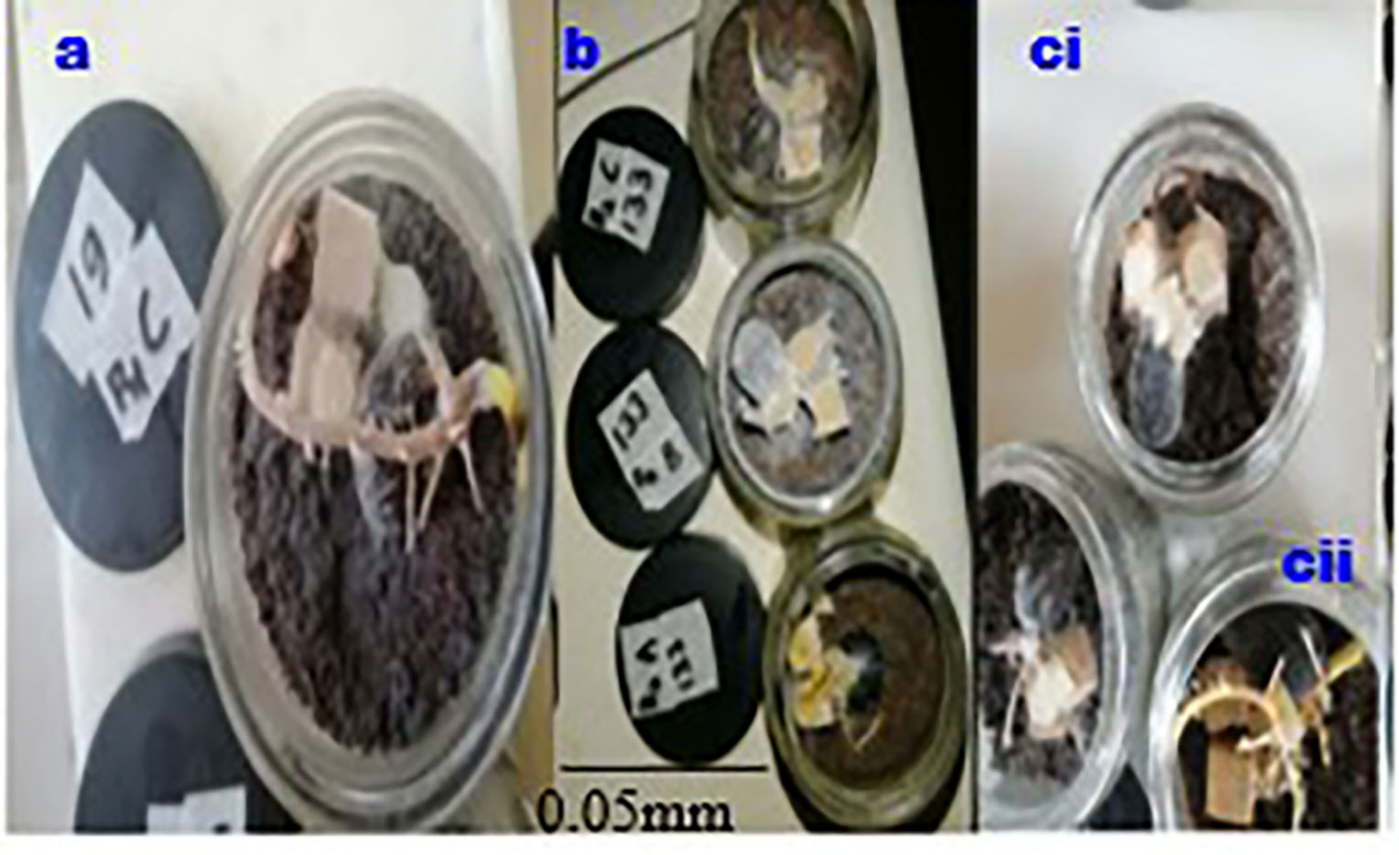
(a, b and ci) Confirmation of the pathogenicity of Ganoderma isolates, cii) control seed nut.

This research presents an *in vitro* artificial inoculation test on oil palm germinated seed nuts, in a controlled environment resulting in the first symptoms development within 2-3 weeks. The first symptoms on inoculated seeds appeared after eight days of inoculation and differences between the inoculates and inoculated germinated seeds and control ( Figure 5cii) became noticeable around 14 to 16 days ( Figure 5aii and Figure 5bii).

After 21 days of artificial inoculation, germinated seed nuts inoculated with WWBs inoculum showed physiological symptoms of disease including root decay ( Figure 5(aii and bii)), necrosis ( Figure 5(aii, and bii)), leaf withering ( Figure 5(aii, and bii)), decaying bole tissue ( Figure 5bii) and other lesions that were absent in the control group of seed nuts ( Figure 5cii).

**3.4.3 External and internal symptoms evaluation**

The percentage infection was determined by considering both external and internal disease indices ( Table 2). The external infections were recorded based on visual symptoms observed in the inoculated seedlings while internal infections were evaluated based on endophytic symptoms analysis and re-confirmation of pathogenicity using molecular tools.

**Table 2.** Statistical analysis of pathogenicity of *Ganoderma* isolates on germinated oil palm seed nuts.

Ganoderma isolates	Estimate Std. Error	t-value	Pr(>|t|)	p-value
T1: GA	72.20000	8.221680	7.6219	0.0000004***
T2: GD _2_B	44.43333	5.059787	5.9914	0.0003237*
T3: TD _2_B	61.10000	6.957682	9.3843	0.0000053**
Control (no inoculum)	13.90000	2.238480	3.6722	2.51120

P-values marked with an asterisk denote the virulence level and the degree of the pathogenic effect of isolated fungi.

**Legend:** Abbreviations: *Std = standard error; *Pr = probability.

### 3.5 Analysis of pathogenicity

Table 2 shows the results of the pathogenicity tests of different *Ganoderma* isolates (T1:GA, T2:GD _2_B, T3:TD _2_B) from BSR-infected oil palms in Ghana. Each isolate (GA, GD _2_B TD _2_B) and the control group were analyzed with estimated effects, standard errors, t-values, and p-values to measure their relative impact on the test seed nuts. Out of 144 seedlings tested, 58.3% were infected. Data showed that GA fungi from the Eastern Region exhibited severe pathogenic effects (t-value: 8.221680) on the germinated seed nuts and caused 26.1% of seedling deaths. Its treatment against the control showed a highly significant (p<0.01) difference with adjusted p-value (p adj) of (0.0000004)***).

**3.5.1 Pathogenicity variation and treatment comparisons**

The treatment GD _2_B isolates showed a significant increase of 44.433 units with a t-value; (5.059787) and a p-value of 0.0003237 compared to the control. Isolates from the Western region (TD _2_B) showed a relatively strong effect (t-value = 6.957682), on the treated seed nuts compared to the control group. GD _2_B shows a decrease of -27.77 difference with a weak pathogenicity effect (p-value: 0.0233249), confidence Interval (lower, upper): (-52.35, -3.19) and a low percentage (2 %) seedling death ( Table 3). Isolate TD _2_B shows a decrease in virulence effect (-11.1 units) compared to the GA isolates, with an adjusted p-value (p adj): of 0.5952326 and a confidence interval (lower and upper): (-35.68, 13.48) of which is not significant. Comparing the pathogenicity effect of TD _2_B and GD _2_B isolates, TD _2_B shows an increase of 16.67 difference with the confidence Interval (lower and upper): (-7.91, 41.25) compared to GD _2_B, but this difference is not significant (p-value = 0.2604245).

**Table 3.** Pathogenicity variation among the *Ganoderma* fungi tested.

Treatment	diff	lwr	upr	p adj
T1(GA)-Control	72.2	47.6207	96.7793	0.00004
T2(GD _2_B)-Control	44.4333	19.854	69.0126	0.00032
T3(TD _2_B) -Control	61.1	36.5207	85.6793	5.3E-06
T2(GD _2_B)-T1(GA)	-27.7767	-52.346	-3.18736	0.02332
T3(TD _2_B) -T1(GA)	-11.1	-35.6793	13.4793	0.59523
T3(TD _2_B)-T2(GD _2_B)	16.6667	-7.91264	41.246	0.26042

**Legend:** Abbreviations: *diff = difference; lwr = lower; upr = upper; p adj = adjustable p-value.

**3.5.2 Symptoms recording**

The highest external symptoms (62.21%) and seedling death were observed in the seed nuts inoculated with GA and TD _2_B fungi, and the lowest severity (4.23 %) was observed in the GD _2_B treated seed nuts. None of the other isolated fungi ( *Trichoderma*, *Xylaria*, *Fusarium* and *Phytophthora* and saprophytes (moulds) produced similar BSR symptoms when tested under Koch’s postulate.

### 3.6 Molecular characterisation and confirmation

**3.6.1 Real-time PCR assay**

**Figure 6.**
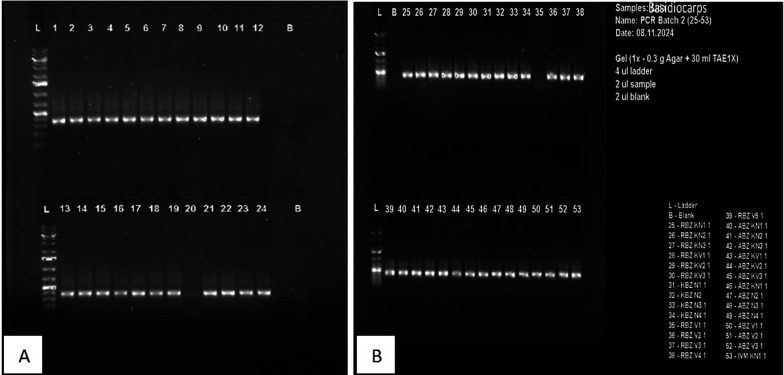
(A) PCR amplify from culture-based isolates and (B) PCR amplification of selected Ganoderma basidiocarps.

A total of 53 samples (24 pure culture-based isolates and 29 Ganoderma Basidiocarps) were amplified. ITS/18rRNA gene sequencing and MLST (multilocus sequence typing) were subsequently used to further characterize and identify the *Ganoderma* fungi isolated from the initial infected samples. DNA extracted from the basidiocarps and the *Ganoderma* isolates from the infected oil palm were amplified using ITS primers pair and a pam clade/GanET primer pair for gene sequencing primer pair. Multilocus sequence typing (MLST) produced a 320 bp fragment and sequenced as the DNA amplicon. A single separation line was observed in Temperature gradient gel Electrophoresis (TGGE) ( Figure 6) at the standard band corresponding to the ITS regions of *Ganoderma* sp. showing consistent results. There was no significant difference between the bands produced by the 29 genotypes of basidiocarps ( Figure 6B) and those of the culture-based samples ( Figure 6A) except for sample numbers (20) and (35) whose bands were very faint.

**3.6.2 Sequence results**

The ITS/ITS4/GanET gene sequence primer pair from the amplified samples showed top matches of >97% to sequences assigned to members of the genus *Ganoderma.* Fully (and validly) named matches of >98% included 99.3% identity to three sequences of *Ganoderma* sp. (HM138671; HM138670 and HM138672) generated from strains assigned to *Ganoderma ryvardenii* (syn. *Ganoderma ryvardense*). Sequence HM138671 is from the type strain of *G. ryvardenii.* (HKAS58053). A match of 99% was also obtained to sequence MN809325 from *Ganoderma wiiroense* strain LDCMY02. Other best matches notified to published strains were only at 92.0-95.6% identity to sequences assigned to G *. boninense.* Out of total sample amplified, 89% of the sequence obtained matches *G. ryvardenii,* 7 % to *G. wiiroense* and 4 % matches *G. boninense.*

**3.6.3 Proof of Koch’s postulate**

**3.6.3.1 Re-isolation and Molecular confirmation of Ganoderma fungi after pathogenicity test**

Amplification of the DNA extracted from the bole and root tissues of the artificially inoculated oil palm seedlings using the ITS1/ITS4 primer pair produced similar base pairs (320 bp) of DNA fragment, which was the same size sequenced as the amplicon of DNA ( Figure 6B) from *Ganoderma* pure culture ( Figure 4). The identity of the fungi was confirmed again as *Ganoderma ryvardenii* via the sequencing of the ITS primer pair amplified products. All the isolates obtained after artificial inoculation had their ITS region sequences that were split into a single line and belonged to the taxon *Ganoderma* which has 99.3% of its gene sequence similarities with the strain of *G. ryvardenii.*

**3.6.4.0 Phylogenetic results**

**3.6.4.1 Analysis of the Phylogenetic tree of *Ganoderma ryvardenii***

The standard band quality of the overall amplification most likely corresponds to a novel species of *Ganoderma.*

**Figure 7.**
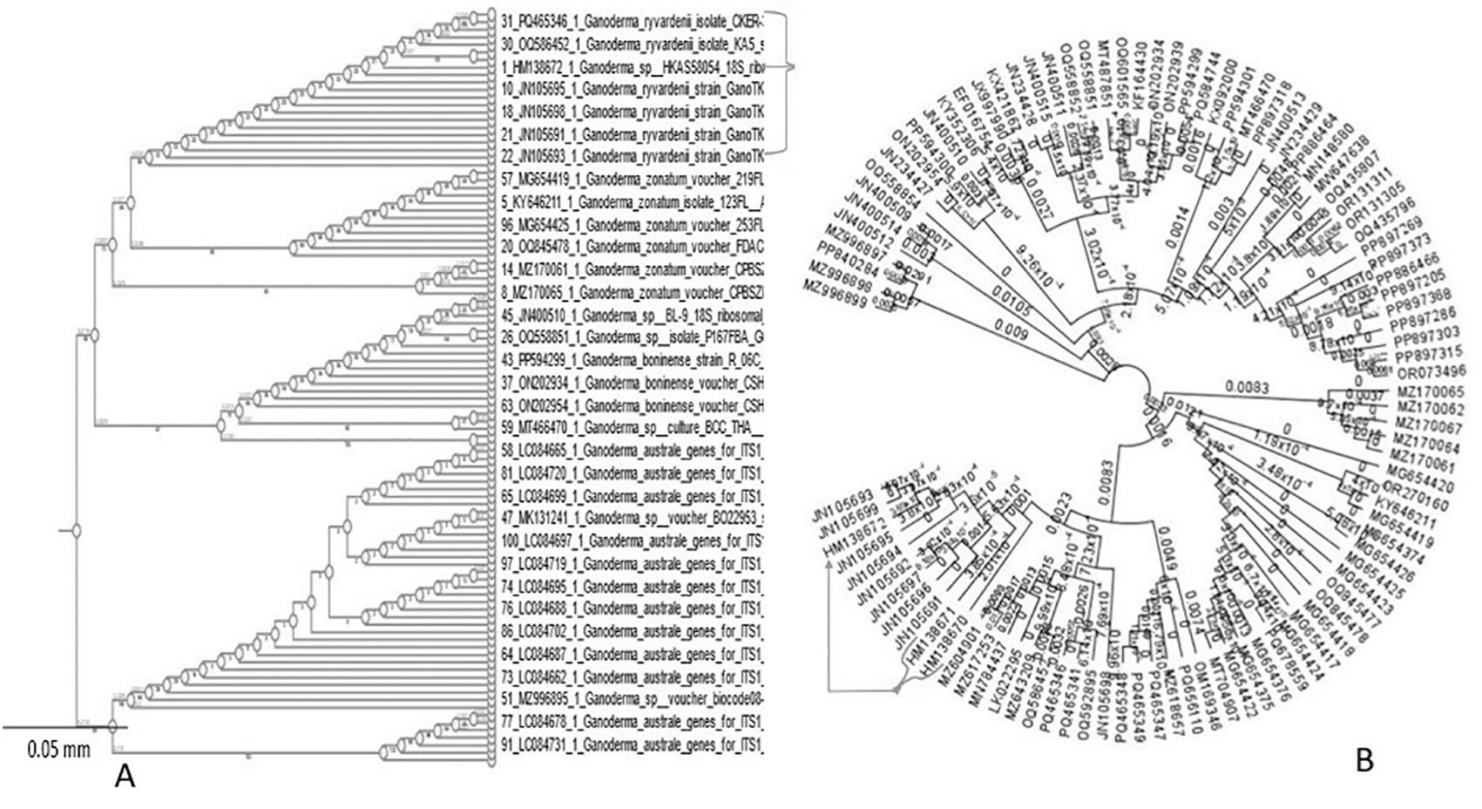
(A) Phylogenetic relationship between different species of Ganoderma fungi associated with BSR on oil palm (B) the accession numbers of the novel species (arrowed) of Ganoderma fungi in this study.

The phylogenetic analyses employed molecular techniques to determine the phylogenetic status of *Ganoderma ryvardenii*. The internal transcribed spacer (ITS) and Ganoderma elongation factor 1-alpha (GanET) sequencing were used along the accession numbers to confirm the identification of a new *Ganoderma* species ( Figure 7B). Sequences were compared using the FASTA software tool and queried against fungal databases of the European Bioinformatics Institute (EBI) and NCBI GenBank. The phylogenetic tree was observed to strictly limit *Ganoderma ryvardenii* as a certain species within the Ganoderma genus, which is relatively and closely related to other BSR-specific species. Majority of the obtained sequences were associated with retrieved sequences within *G. ryvardenii* (indicated by the arrows in Figure 7B), confirming its distinct lineage within the genus *Ganoderma.*

This further confirmed the close affinity of *G. ryvardenii* with other BSR-infecting *Ganoderma* species of oil palms. Clustering observed in ( Figure 7A and B, arrowed) suggests common evolutionary ancestry and probable identical pathogenic mechanisms in such species.

## 4. Discussion

### 4.1 Field observation and percentage infection

Field observations and percentage infection rates of *Ganoderma* in the selected oil palm plantations provide important information about the disease’s spread, effects, and mechanism of transmission. Although BSR was initially found in palms older than 25–30 years old, it has recently been reported that the disease can affect younger palms, including those as young as one year old ( Zakaria, 2023).

This study showed a higher infection rate in the Eastern region than in the other two Regions surveyed. Despite the low disease incidence in the selected districts compared with the total number of palm trees analysed, the rate of disease spread was of grave concern. A similar observation was made by Lekete-Lawson et al. (2024) in a separate study on oil palm in Ghana.

### 4.2 Physiological characteristics of Ganoderma symptoms

The physiological features portrayed by the pathogen in this study, shared similar characteristics of *Ganoderma* species described by Bharudin et al. (2022) and Chong et al. (2017). According to these authors, basal stem rot (BSR) in oil palm is a disease caused by bracket fungi, producing basidiocarps which are perennial hard-knot and pinhead-like when immature; fan-shaped and large when mature; with irregular margins/blades (lamellae/gills). They are called *Ganoderma* fungi and are members of the phylum Basidiomycota and the family *Ganodermataceae.* Hushiarian et al. (2013) and Pilotti (2005) also reiterated that the basidiocarp is a typical characteristic feature of *Ganoderma* fungi. These basidiocarps are lignicolous and leathery and sometimes grow in a hoof-like form on the trunks of infected palms, just as observed in the present study ( Figure 2). Some of the heavily infected palms that were seen full of basidiocarps were rotten, fractured and collapsed as a result of the infection. Similarly, Siddiqui et al. (2021) and Josephin et al. (2024) observed that after the oil palm tree becomes infected, the fungus quickly spreads throughout the trunk tissues, causing the internal structures to deteriorate leading to the collapse and the death of the infected palms.

### 4.3 Isolation and identification of causal agent(s) of the BSR disease on oil palm

**4.3.1 Macromorphological characteristic of culture of *Ganoderma* fungi**

The isolated fungi were grouped based on their macromorphological characteristics and micrograph features. They were further ranked based on their taxonomy and percentage of occurrences. Research has shown that most *Ganoderma* species identified in the past were based on morphological characteristics ( Latiffah & Ho, 2005). However, studies by Mohd Rakib et al. (2014) showed that *Ganoderma* species are genetically heterogeneous and different based on their geographical origin, thus resulting in genetic variations even within the same species ( Hong et al., 2001). This was evident in the present study when the macromorphological characteristics of colonies of *Ganoderma* fungi isolated from the three regions appeared different.

The study showed slight differences in culture characteristics among the three *Ganoderma* isolates (GA, GD _2_B and TD _2_B) regarding their colony colour, texture and growth rate. The white and stranded mycelium, velvety with concentric cottony and hyphal-knot structure produced by GD _2_B isolates were similar to what was found in the studies conducted by Beck et al. (2018) and Badalyan et al. (2015) on several *Ganoderma* species, in which some species of *Ganoderma* appeared as cottony texture with powdery radiating parallel hyphae which were white and later changed to lemon yellowish. TD _2_B appeared white, fluffy, cottony and velvety but turned yellowish-brown as it progressed. Mohanty et al. (2011) discovered that some *Ganoderma* species age as their colonies turn yellowish to brown.

The varied growth rate observed among the isolates with GA exhibiting the fastest growth rate could depend on several variables, including the culture’s duration, the media’s composition, the ambient temperature and the media composition. According to Gáperová and Petrýdesová (2009) several elements, including light, temperature, medium volume, nutritional component ratio, and others, might influence the properties of mycelia under study. Although our findings suggest that the macromorphological traits of cultures and the mycelium’s growth rate may be utilised to identify specific *Ganoderma* species, more research is needed to distinguish individual species.

### 4.4 Confirmation of pathogenicity test of isolated fungi

**4.4.1 Source of inoculum**

*Triplochiton scleroxylon* (Wawa woodblocks-(WWBs)) used for inoculum in this study helped to retain the survival of isolated fungi for the inoculation. The studied Ganoderma fungi (GA, TD _2_B and GD _2_B) were able to colonise the inoculated WWBs but at varied degrees of myceliation. According to Mangaiha et al. (2019), woody debris (inoculum) plays an important role in the long-term survival of Ganoderma fungi. They enhance the resistance of the growth of *Ganoderma* fungi against environmental stress ( Loyd et al., 2018). Woodblocks (WWBs), as a source of inoculum, produced successful infection on oil palm germinated seeds.

Although, research revealed that Rubberwood block (RWB) is an ideal substrate that improves the long-term survival, biological yield and productivity of Ganoderma fungi ( Breton et al. (2006); Alexander, (2017) and Nusaibah et al. (2016)), however, an experiment conducted by Angel et al., (2021) showed that if RWB becomes scarce, other substrates, including sawdust and wood chippings, could be used as a source of inoculum, through non-invasive tissue culture techniques. This was confirmed in this study when Wawa woodblock (WWB) as a substrate and inoculum source, was able to establish Ganoderma infection on the oil palm germinated seed nuts marking the first successful attempt at using the tissue culture technique for establishing *Ganoderma* infections on oil palm germinated seed nuts in Ghana. Nonetheless, other isolated fungi such as *Trichoderma*, *Phytophthora, Fusarium*, *Xylaria, and* others (moulds) were not able to colonise the substrate (WWBs).

### 4.5 *In vitro* artificial inoculation

Breton et al *.* (2005, 2006) showed a positive correlation between germinated seeds and 3-month-old seedlings in the discrimination between progenies for their susceptibility to *Ganoderma.* Although the formation of Ganoderma basidiocarps was not observed in this experiment probably due to the short time allocation and the substrate used, the disease symptoms, displayed by GA, GD2B and TD2B isolates were similar to those of a typical BSR disease in young seedlings. Similar features were also observed by Angel et al. (2021) after post-inoculation of plantlets with sawdust inoculum which displayed physiological symptoms of the disease that resembled those of a typical BSR infection. Unlike inoculation tests on 3-month-old seedlings that had been conducted by previous researchers including Idris *et al.* (2004) and Rees *et al*. (2007), the results of this study have shown that *in vitro* inoculation test can be used as an alternative to reduce the age needed for inoculation techniques, shortening inoculation duration, and ensure consistency in experimental results.

### 4.6 Analysis of Pathogenicity

**4.6.1 Pathogenicity variation**

The results suggest robust evidence against the null hypothesis, indicating that all isolates tested in this study exhibit a higher degree of pathogenicity. However, some disparities were observed in the aggressiveness of isolated *Ganoderma* fungi from the Eastern, Western, and Central regions of Ghana comparing their pathogenicity against control. GA isolate has the most virulent pathogenic effect causing the highest disease infection and death of the test seed nuts. This points to the fact that GA is likely responsible for the majority of BSR infections in the selected oil palm plantations in Ghana. The variations in the level of pathogenicity identified among the Ganoderma isolates in this study correspond to findings by Lo et al. (2023), Goh et al. (2014), Mohd Rakib et al. (2014) and Breton et al. (2006). These individual authors also observed some differences in the degree of pathogenicity of Ganoderma pathogens on oil palms from different geographical locations. For instance, Lo et al. (2023) and Mohd Rakib et al. (2014), observed that there were some variations in aggressiveness within the species of *Ganoderma boninense* obtained from infected oil palms at different estates in Sarawak.

Breton et al. (2006) discovered that *G. boninense* isolates from different plantations in Indonesia had varying levels of aggressiveness. Likewise, in Malaysia, the transmission rate of *Ganoderma* disease varies by estate, potentially indicating differences in *G. boninense* aggressiveness across isolates from different regions ( Goh et al., 2014). Mohd Rakib et al. (2014) also revealed similar observations about the significant variation in the aggressiveness levels of *Ganoderma* species isolated from different areas. The authors observed that *G. boninense* and *G. zonatum* isolated from Betong and Miri differed in their aggressiveness. This was demonstrated when all the Ganoderma isolates obtained from various oil palm growing areas in Ghana showed varied responsiveness in their disease establishment.

The gradual decline of growth among the germinated seed nuts after being inoculated with *Ganoderma* isolates was observed. From day 14, the virulence factor of GA isolates from the Eastern region could be clearly distinguished from the others (TD _2_B and GD _2_B). The effect of the isolated fungi appeared after 15 days of incubation with initial infection of lesions forming on the roots of the inoculated seed nuts. This corresponds with the characteristic infection pattern by *Ganoderma* spp. as reported by Rees et al. (2007) and Breton et al. (2006). According to Rees et al. (2007), Ganoderma infection usually starts from the root system of the infected palms when in direct contact with the inoculum. Isolate GA has the strongest pathogenic effect on the test seed nuts, compared to isolate TD _2_B. However, the pathogenic effect of TD _2_B isolate is still highly significant (p<0.05).

The highest external symptoms and seedling death was observed in the seed nuts inoculated with GA and TD _2_B fungi whereas the lowest severity was observed in the GD _2_B treated seed nuts. Apparently, none of the other isolated fungi ( *Trichoderma*, *Xylaria*, *Fusarium* and *Phytophthora* and saprophytes (moulds) were able to produce similar BSR symptoms observed on the original samples when tested under Koch’s postulate.

### 4.7 Molecular characterisation and confirmation

**4.7.1 Real-time PCR assay**

Despite the weak fragment shown in the band formation of samples 20 and 35, probably due to PCR inhibitors, the standard band quality of the overall amplification most likely corresponds to a novel species of *Ganoderma.* He et al., (2022) predicted that approximately 1.4 to 4.2 million species of Basidiomycota will be discovered worldwide by 2030, as over 54,000 have already been reported. Since the coming of the molecular age, several species previously described morphologically are now redefined as new taxa in addition to the finding of new taxa. To this effect, there are now over 36,000 Basidiomycota species, according to a comprehensive systematic survey using molecular data ( Begerow et al., 2018).

The molecular tools employed in this experiment allowed us to differentiate between numerous fungal species of Ganoderma disease that are present in Ghana’s oil palm fields. Sequences of other phylogenetic markers useful in the phylogeny of *Ganoderma* fungi supported our hypothesis that the isolates most likely represent a novel species of *Ganoderma.* For instance, the majority of the ITS sequences of *Ganoderma* sp. [HM138671; HM138670 and HM138672] generated from strains assigned to *Ganoderma ryvardenii* (syn. Ganoderma ryvardense) HM138671; HM138670 and HM138672 published in Mycosphere 2 (2): 179–188. Of these, sequence HM138671 is from the type of strain of *G. ryvardenii.* (HKAS58053). There was a single match of 100% to sequence MN809325 from *Ganoderma wiiroense* strain LDCMY02 but this has not been published in a peer-reviewed publication and is quite distinct from other sequences of *G. wiiroense* in GenBank, so it is discounted here. Thereafter, the third-best matches to published strains were also identified to sequences assigned to *G. boninense.*

Additionally, the sequence alignment in phylogenetic tree building also revealed high similarities among Ganoderma strains identified in Cameroon (HKAS58053) and the new strains identified in Ghana (HM138671; HM138670 and HM138672). As such the phylogenetic analyses based on ITS sequences and their accession numbers confirmed the novelty of this *Ganoderma* species on oil palm in Ghana. This finding enhances our understanding of the diversity and distribution of specific *Ganoderma* species within African mycobiota. This also confirms the initial discovery made by Kinge and Mih (2011), who first reported the pathogen on oil palm in Cameroon and gave the species name as *Ganoderma ryvardense* with the specific epithet in honour of Lief Ryvarden, a distinguished mycologist who has contributed significantly to genus *Ganoderma* and African mycobiota. (Mycosphere 2 (2): 179–188). Evidently, this is the first report of *G. ryvardenii* causing BSR disease on oil palm in Ghana and possibly first in West Africa but second in Africa.

## 5. Conclusion

The study uses molecular techniques to identify and confirm the Ganoderma species responsible for causing BSR disease in selected oil palm plantations in Ghana.

The sequences of other phylogenetic markers useful in identifying and confirming *Ganoderma* fungi supported our hypothesis that these isolates represent novel species of *Ganoderma.*

Thus, this study underscores the importance of using molecular techniques for accurate identification. It highlights the genetic diversity and morphological variability of *Ganoderma* species on oil palm in Ghana forming a crucial understanding of their role in causing BSR disease on oil palm.

The fungus was first identified as *Ganoderma* sp. based on the physiological features (Fruiting bodies/basidiocarps), disease symptoms, culture morphological characteristics and later confirmed through Koch’s postulate, molecular amplification of internal transcribed spacer (ITS sequence) and basidiomycete-selective primer (GanET) to obviate the potential co-amplification of contaminant non-basidiomycetous fungi.

As such, these findings provide baseline information for future studies regarding the emergence of new species of *Ganoderma* on oil palm in Ghana.

## Data Availability

Figshare: Report on Basal Stem Rot (BSR) disease on oil palm in Ghana, DOI: https://doi.org/10.6084/m9.figshare.29382632 ( Lekete-Lawson, 2025). The project contains following underlying data:
• Extracted files • Gano data analysis. 1R • Book 1 Gano. Raw. Data are available under the terms of the Creative Commons Attribution 4.0 International license (CC-BY 4.0). The data generated in this study have been deposited in the Figshare repository and can be accessed at [DOI: 10.6084/m9.figshare.29382632]. Some of the article’s data (Accession numbers) can also be found in the following Nucleotide database [NCBI Blast: gb/HM138670/ https://doi.org/10.6084/m9.figshare.29382632], others were retrieved from the following public domain resources: The dataset includes raw sequencing data, processed data and Metadata (Map of districts surveyed, Treatment data on pathogenicity, R-Software data analysis and Ganoderma molecular work). The data are licensed under a CC-BY 4.0 license, which permits unrestricted use, distribution, and reproduction in any medium, provided the original author and source are credited. Further details are available from the corresponding author upon request through the link: https://figshare.com/authors/Emmanuellah_Lekete-Lawson/20618918. https://doi.org/10.6084/m9.figshare.29382632. Figshare: Data availability on the first report of *G. ryvadenii* causing BSR disease on oil palm in Ghana (update), DOI: https://doi.org/10.6084/m9.figshare.29382632 ( Lekete-Lawson, 2025). The project contains the following extended data:
• jpg Districts surveyed in the course of study • sequences used 1 • Accession numbers retrieved • sequence blast doc. TGCGGAAGGATCATTATC • NCBI Blast_gb_HM138670_ Data are available under the terms of the Creative Commons Attribution 4.0 International license (CC-BY 4.0).
