# Supplementary material for: First Report of Ganoderma ryvardenii causing Basal Stem Rot (BSR) disease on oil palm ( Elaeis guineensis Jacq.) in Ghana
Source: F1000Res. 2025 Jul 7;14:413. Originally published 2025 Apr 8. [Version 2] doi: 10.12688/f1000research.161972.2 (PMC12125568; doi:10.12688/f1000research.161972.2)
Supplement: Peer review history

# Reviewer report: Reviewer response for version 2

**DOI:** 10.5256/f1000research.184059.r397250

**Published:** 2025-07-25

**Authors:** Supriyanto Supriyanto

**Affiliations:** 1 Tanjungpura University, Jl. Prof. Dr. H. Hadari Nawawi, Pontianak, Indonesia

The manuscript has been well revised, but there are still several sections that have not been adjusted.

 These sections are:

 1. Method (3.2). The authors have been changed to "Morphological characteristics....", but the results and discussion section (4.2) still uses the phrase "Physiological characteristics...."; this should be adjusted.

 2. In the results and discussion. In Figure 2. "(B) Initial stage of infection and pinhead of the Ganoderma mushroom" is an inappropriate statement. It would be better to delete the phrase "Initial stage of infection" and use just the phrase "pinhead of the Ganoderma mushroom" because in this article, there is no evidence that the pinhead is the initial infection of Ganoderma. Researchers all state that the initial infection is more often through root contact.

 Furthermore, it would be better not to use the term "Ganoderma mushroom"; it would be more appropriate to use the term Ganoderma fruiting body or carpophore, which researchers commonly use.

Is the work clearly and accurately presented and does it cite the current literature?

Yes

If applicable, is the statistical analysis and its interpretation appropriate?

Partly

Are all the source data underlying the results available to ensure full reproducibility?

Yes

Is the study design appropriate and is the work technically sound?

Yes

Are the conclusions drawn adequately supported by the results?

Yes

Are sufficient details of methods and analysis provided to allow replication by others?

Partly

Reviewer Expertise:

Plantation crop cultivation, plant disease, oil palm disease, and biological control of plant disease.

I confirm that I have read this submission and believe that I have an appropriate level of expertise to confirm that it is of an acceptable scientific standard.

# Reviewer report: Reviewer response for version 1

**DOI:** 10.5256/f1000research.178088.r380554

**Published:** 2025-05-30

**Authors:** Lisnawita Lisnawita

**Affiliations:** 1 Universitas Sumatera Utara,, Padang Bulan, Indonesia

In the method, it is not written how many oil palm plants were observed in the field research, but in the result is written 3000 oil palm trees were assessed.

 Figure 2, 4, 5, 6 are not clear

 Microscopic observations should be accompanied by Ganoderma spore images

 Researchers only used ITS and GanET for molecular identification. It is recommended to add additional genetic markers such as TEF1α or RPB2 for deeper validation, because the genus Ganoderma is known to have taxonomic complexity

 Conclusions should be stated explicitly. Do not state that the isolate is “probably” a new species.

Is the work clearly and accurately presented and does it cite the current literature?

Yes

If applicable, is the statistical analysis and its interpretation appropriate?

Yes

Are all the source data underlying the results available to ensure full reproducibility?

Yes

Is the study design appropriate and is the work technically sound?

Yes

Are the conclusions drawn adequately supported by the results?

Yes

Are sufficient details of methods and analysis provided to allow replication by others?

Yes

Reviewer Expertise:

Phytopathology, Nematology

I confirm that I have read this submission and believe that I have an appropriate level of expertise to confirm that it is of an acceptable scientific standard.

# Reviewer report: Reviewer response for version 1

**DOI:** 10.5256/f1000research.178088.r380559

**Published:** 2025-05-19

**Authors:** Efi Toding Tondok

**Affiliations:** 1 Plant Protection, IPB University, Bogor, West Java, Indonesia

Method.

 1)Scores on disease severity must be written clearly.

 2) This is Basidiomycota, so you will never find spores by mounting mycelia on object glass.

Result.

 1) Please add basidiospores morphometrics as one of the important features for morphological identification.

 The 0.5 mm bar is too long for microscopic measurement.

 2) Fig 5 and 6: please put the objects in a good arrangement, so the reader will be easy to understand. Don't forget the control. The 0.5 mm bar is very annoying.

 3) It is very important to include pictures from the fields, the mild or early symptom to severe or advance symptom. Formation of basidiocarp indicating the advance symptom, plant almost die. 

 4) How many species of Ganoderma found in Ghana?

 5) Please put the same codes on your isolates since morphological identification - pathogenicity tests - molecular identification: easy to track it, to prove that the tested isolate is the same as the isolate identified morphologically and molecularly 

Discussion.

 Please explain why disease incidence and disease severity are highest in K29-B, compared to other places

Conclusion. 

 To prove that the primers you use can distinguish Ganoderma well, you need to test them on several Ganoderma species of known species. So, please go only to your finding.

Is the work clearly and accurately presented and does it cite the current literature?

Yes

If applicable, is the statistical analysis and its interpretation appropriate?

Partly

Are all the source data underlying the results available to ensure full reproducibility?

Yes

Is the study design appropriate and is the work technically sound?

Yes

Are the conclusions drawn adequately supported by the results?

Partly

Are sufficient details of methods and analysis provided to allow replication by others?

Partly

Reviewer Expertise:

Phytopathology, Mycology, Seed Pathology, Biological Control

I confirm that I have read this submission and believe that I have an appropriate level of expertise to confirm that it is of an acceptable scientific standard, however I have significant reservations, as outlined above.

# Reviewer report: Reviewer response for version 1

**DOI:** 10.5256/f1000research.178088.r377597

**Published:** 2025-04-30

**Authors:** Supriyanto Supriyanto

**Affiliations:** 1 Tanjungpura University, Jl. Prof. Dr. H. Hadari Nawawi, Pontianak, Indonesia

Overall, this article is well written. However, some parts need to be strengthened to make it a reliable reference. 

 1. In the last paragraph of the introduction, considering the author's claim about the discovery of a new species of Ganoderma that causes Basal Stem Rot disease in oil palm, it is better to add a new, more complete reference about reports related to the types of Ganoderma that previous researchers have reported.

 2. Method. Observation of disease severity in oil palm in the field uses a score of 0 to 4. The scoring criteria should be displayed in full. In addition, the use of the number of fruiting bodies as an indicator of severity seems rather risky because there has been no report on this. Based on experience in Indonesia, the number of fruiting bodies that can be found is not directly related to the severity of the disease. 

 3. Method, pathogenicity test. To be clearer, it is necessary to further describe how to arrange RWB, oil palm seeds, and the media used in the container. Is RWb mixed with the media, or how?

 4. Results, point 3.2. This subtitle is a bit inappropriate, because the content is a description of the morphology of the Ganoderma fruit body found. It would be better if the title were "Morphological characteristic......"

 5. Results, Figure 2. In part (B), it is stated as the Initial stage of infection and the pinhead of the Ganoderma mushroom. This is a less appropriate statement, because the initial infection of Ganoderma on oil palm is not related to the appearance of pinheads. The appearance of pinheads is a sign that the infection is advanced and the disease is severe. 

 6. Figure 5. The image is not clear. The 0.5 mm line mark does not seem to match the original condition. In part (ii), root decay, necrosis, and decaying bole tissue are not visible. If possible, the image can be displayed at a closer up, so that the message to be conveyed can be better received by the reader. 

 7. Table 2. If possible, it would be better if the disease severity data could be displayed.

 8. Figure 6. Not necessary. This image shows nothing.

 9. It would be better if a general discussion were added that supports the main findings. For example, the condition of the environmental vegetation around the sample plantation is suspected of causing G. ryvardenii to be there and attack oil palms.

Is the work clearly and accurately presented and does it cite the current literature?

Yes

If applicable, is the statistical analysis and its interpretation appropriate?

Partly

Are all the source data underlying the results available to ensure full reproducibility?

Yes

Is the study design appropriate and is the work technically sound?

Yes

Are the conclusions drawn adequately supported by the results?

Yes

Are sufficient details of methods and analysis provided to allow replication by others?

Partly

Reviewer Expertise:

Plantation crop cultivation, plant disease, oil palm disease, and biological control of plant disease.

I confirm that I have read this submission and believe that I have an appropriate level of expertise to confirm that it is of an acceptable scientific standard, however I have significant reservations, as outlined above.
